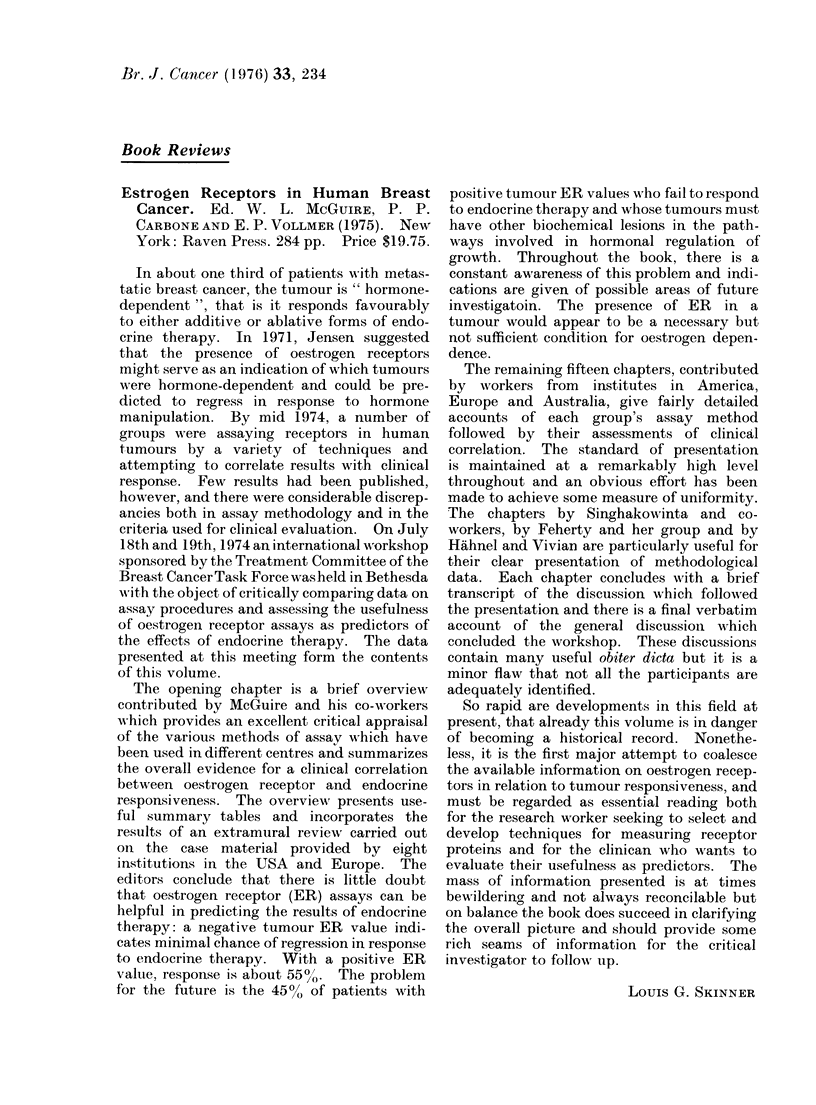# Estrogen Receptors in Human Breast Cancer

**Published:** 1976-02

**Authors:** Louis G. Skinner


*Br. J. Cancer* (1976) **33**, 234

### *Book Reviews*

**Estrogen Receptors in Human Breast Cancer.** Ed. W. L. McGuire, P. P. Carbone and E. P. Vollmer (1975). New York: Raven Press. 284 pp. Price $19.75.

In about one third of patients with metastatic breast cancer, the tumour is " hormone-dependent ", that is it responds favourably to either additive or ablative forms of endocrine therapy. In 1971, Jensen suggested that the presence of oestrogen receptors might serve as an indication of which tumours were hormone-dependent and could be predicted to regress in response to hormone manipulation. By mid 1974, a number of groups were assaying receptors in human tumours by a variety of techniques and attempting to correlate results with clinical response. Few results had been published, however, and there were considerable discrepancies both in assay methodology and in the criteria used for clinical evaluation. On July 18th and 19th, 1974 an international workshop sponsored by the Treatment Committee of the Breast Cancer Task Force was held in Bethesda with the object of critically comparing data on assay procedures and assessing the usefulness of oestrogen receptor assays as predictors of the effects of endocrine therapy. The data presented at this meeting form the contents of this volume.

The opening chapter is a brief overview contributed by McGuire and his co-workers which provides an excellent critical appraisal of the various methods of assay which have been used in different centres and summarizes the overall evidence for a clinical correlation between oestrogen receptor and endocrine responsiveness. The overview presents useful summary tables and incorporates the results of an extramural review carried out on the case material provided by eight institutions in the USA and Europe. The editors conclude that there is little doubt that oestrogen receptor (ER) assays can be helpful in predicting the results of endocrine therapy: a negative tumour ER value indicates minimal chance of regression in response to endocrine therapy. With a positive ER value, response is about 55%. The problem for the future is the 45% of patients with positive tumour ER values who fail to respond to endocrine therapy and whose tumours must have other biochemical lesions in the pathways involved in hormonal regulation of growth. Throughout the book, there is a constant awareness of this problem and indications are given of possible areas of future investigatoin. The presence of ER in a tumour would appear to be a necessary but not sufficient condition for oestrogen dependence.

The remaining fifteen chapters, contributed by workers from institutes in America, Europe and Australia, give fairly detailed accounts of each group's assay method followed by their assessments of clinical correlation. The standard of presentation is maintained at a remarkably high level throughout and an obvious effort has been made to achieve some measure of uniformity. The chapters by Singhakowinta and co-workers, by Feherty and her group and by Hähnel and Vivian are particularly useful for their clear presentation of methodological data. Each chapter concludes with a brief transcript of the discussion which followed the presentation and there is a final verbatim account of the general discussion which concluded the workshop. These discussions contain many useful *obiter dicta* but it is a minor flaw that not all the participants are adequately identified.

So rapid are developments in this field at present, that already this volume is in danger of becoming a historical record. Nonetheless, it is the first major attempt to coalesce the available information on oestrogen receptors in relation to tumour responsiveness, and must be regarded as essential reading both for the research worker seeking to select and develop techniques for measuring receptor proteins and for the clinican who wants to evaluate their usefulness as predictors. The mass of information presented is at times bewildering and not always reconcilable but on balance the book does succeed in clarifying the overall picture and should provide some rich seams of information for the critical investigator to follow up.

Louis G. Skinner